# A four-microRNA classifier as a novel prognostic marker for tumor recurrence in stage II colon cancer

**DOI:** 10.1038/s41598-018-24519-4

**Published:** 2018-04-18

**Authors:** Havjin Jacob, Luka Stanisavljevic, Kristian Eeg Storli, Kjersti E. Hestetun, Olav Dahl, Mette P. Myklebust

**Affiliations:** 1 Department of Clinical Science, Faculty of Medicine, University of Bergen, Bergen, Norway; 2 Department of Oncology and Medical Physics, Haukeland University Hospital, Bergen, Norway; 3 Department of Surgery, Haraldsplass Deaconess Hospital, Bergen, Norway

## Abstract

About 20 percent of TNM-stage II colon cancer patients who are treated by surgical resection develop recurrence, and adjuvant chemotherapy in this group is still debated among researchers and clinicians. Currently, adverse histopathological and clinical factors are used to select patients for adjuvant chemotherapy following surgery. However, additional biomarkers to classify patients at risk of recurrence are needed. We have conducted a study using fresh frozen tumor tissue from 54 TNM-stage II colon cancer patients and performed microRNA profiling using next-generation sequencing. For the selection of the prognostic microRNAs, a LASSO Cox Regression model was employed. For the validation, we used the publically available TCGA-COAD cohort (n = 122). A prognostic panel of four micorRNAs (hsa-miR-5010-3p, hsa-miR-5100, hsa-miR-656-3p and hsa-miR-671-3p) was identified in the study cohort and validated in the TCGA-COAD cohort. The four-microRNA classifier successfully identified high-risk patients in the study cohort (P < 0.001) and the validation cohort (P = 0.005). Additionally, a number of established risk factors and the four-miRNA classifier were used to construct a nomogram to evaluate risk of recurrence. We identified a four-microRNA classifier in patients with TNM-stage II colon cancer that can be used to discriminate between patients at low- and high risk of recurrence.

## Introduction

Colon cancer is one of the most common cancers worldwide and is the second most common cancer in developed countries^1,2^. Surgery and chemotherapy are the most common treatments for colon cancer patients, and the TNM (Tumor-Node -Metastasis) - staging system has been widely used for colon cancer management allocation clinically^3^. Adjuvant chemotherapy after surgery is a standard regimen for patients with TNM-stage III to improve survival and reduce the risk of tumor recurrence^4^. In contrast, the benefit of adjuvant chemotherapy for TNM-stage II patients is still debated, and a quarter of these patients die from tumor recurrence after surgery^5–8^. Currently, clinicopathological factors do not clearly guide patients‘ prognostic stratification, nor do they identify the patients with benefit from adjuvant chemotherapy in TNM-stage II colon cancer^9,10^.

One of the most recently established prognostic factors in early stages of colon cancer is a defective DNA mismatch repair (MMR) system in the tumor cells. Failure of the MMR system can be tested by immunohistochemistry for loss of MMR proteins, or by testing for microsatellite instability (MSI) by PCR. A study based on a large series of unselected cases found that immunohistochemistry and microsatellite instability are comparable methodologies for detecting defective MMR system^11^. The prevalence of MMR system deficiency is higher in early stages (20% in stage I-II) and it is associated with better survival in TNM-stage II colon cancer^12–18^. Importantly, it is known that patients in TNM-stage II with MSI does not benefit from adjuvant 5-flurouracil based chemotherapy^19–21^. Other established clinicopathological risk factors for TNM-stage II colon cancer patients are pT4 and low total number of lymph nodes removed^22,23^. Unfortunately, these factors are not able to successfully identify all patients at high risk of recurrence. Consequently, there is a need for biomarkers to add prognostic value to the traditional clinicopathological factors and to guide the clinicians to discriminate high-risk and low-risk patients. As a result of the modest therapeutic benefit of adjuvant chemotherapy in TNM-stage II colon cancers, prognostic biomarkers have a major relevance.

Recently, microRNAs (miRNAs) are identified as prognostic biomarkers in TNM-stage II colon cancer patients, such as miR-21, miR-29a, miR-106a, miR-556^24–27^. A study identified a classifier based on six-miRNAs as a prognostic biomarker in TNM-stage II colon cancer patients^28^. Another study aimed to reproduce this study, identified three of these miRNAs as the best prognostic marker^29^. In our previous study, we identified a prognostic 16-miRNA signature among 83 selected miRNAs in TNM-stage II and III colon cancer patients^30^. These studies have used microarrays and quantitative real-time PCR (RT-qPCR).

In order to identify a prognostic biomarker with the ability to classify TNM-stage II patients at high- and low- risk of recurrence, we here investigated global miRNA expression profiling using next-generation sequencing (NGS).

## Results

### Cohorts

A summary of the demographical features for the 54 patients in the study (HDH-CC) cohort and the 122 patients in the validation (TCGA-COAD) cohort is shown in the Table 1. The median age of the HDH-CC cohort was 73 years. The patients of the TCGA-COAD cohort were younger (median age 67.8 years). There was no difference between fraction of women and men in the HDH-CC cohort (52% women and 48% men) and in the TCGA-COAD cohort (57% men and 43% women). In the HDH-CC cohort, 65% of the patients had proficient MMR status, and 59% of the TCGA-COAD cohort had MSS. MSI-status is used instead of MMR-status in the validation cohort because immunohistochemistry for the MMR proteins was not available for all the patients. Eighty five percent of the patients had well or moderate differentiated tumors and 93% had adenocarcinoma in the HDH-CC cohort. Information regarding tumor differentiation status was not available for the TCGA-COAD cohort. A total of 13% and 19% of the patients had recurrence in the HDH-CC cohort and TCGA-COAD cohort, respectively. The association between recurrence and the four-miRNA classifier was statistically significant (P < 0.001 and P = 0.006, respectively). The relation between tumor stage and the four-miRNA classifier was statistically significant in the HDH-CC cohort (P = 0.04). There were no statistically significant associations between the four-miRNA classifier and the other clinicopathological variables (Table 1).

**Table 1:** Baseline characteristics of patients and their association with four-miRNA classifier.

	HDH-CC (n = 54)				TCGA-COAD (n = 122)			
	Number of patients	Low risk (%)	High risk (%)	p-value*	Number of patients	Low risk (%)	High risk (%)	p-value*
Age				0.74				0.96
≤72.98	22	18 (82%)	4 (18%)		53	34 (64%)	19 (36%)	
>72.98	32	25 (78%)	7 (22%)		69	44 (64%)	25 (36%)	
Gender				0.38				0.93
Women	28	21 (75%)	7 (25%)		52	33 (64%)	19 (36%)	
Men	26	22 (85%)	4 (15%)		70	45 (64%)	25 (36%)	
MMR status				0.44				
Deficient	15	13 (87%)	2 (13%)		—	—	—	
Proficient	35	27 (77%)	8 (23%)		—	—	—	
ND	4	—	—		—	—	—	
MSI status								0.63
MSI	—	—	—		50	33 (66%)	17 (34%)	
MSS	—	—	—		72	45 (62%)	27 (38%)	
Tumor stage				0.04				0.67
T3	51	42 (82%)	9 (18%)		115	73 (64%)	42 (36%)	
T4	3	1 (33%)	2 (67%)		7	5 (71%)	2 (29%)	
Differentiation				0.19				
Well/Moderate	46	38 (83%)	8 (17%)		—	—	—	
Poor	8	5 (63%)	3 (37%)		—	—	—	
TLN				0.23				0.4
<12	5	5 (100%)	0 (0%)		13	11 (85%)	2 (15%)	
≥12	49	38 (78%)	11 (22%)		96	60 (63%)	36 (37%)	
ND	—	—	—		13	—	—	
Histology type				0.12				
Adenocarcinoma	50	41 (82%)	9 (18%)		—	—	—	
Variant^a^	4	2 (50%)	2 (50%)		—	—	—	
Recurrence				<0.001				0.006
Yes	7	1 (14%)	6 (86%)		23	9 (39%)	14 (61%)	
No	47	42 (89%)	5 (11%)		99	69 (70%)	30 (30%)	

^a^Variant includes signet ring and mucinous carcinoma, ND = not determined, TLN = total lymph node.

### Identification and construction of the four-miRNA classifier

In order to find the prognostic miRNAs in the HDH-CC cohort, we assessed TNM-stage II colon cancer patients using a LASSO Cox regression analysis model. A panel of four miRNAs (hsa-miR-5010-3p, hsa-miR-5100, hsa-miR-656-3p and hsa-miR-671-3p) was found to be the best predictor of recurrence in TNM-stage II colon cancer patients. The dichotomized miRNA expression values weighted by the coefficients from the LASSO regression generated a prognostic index for each patient using the formula where *E-miR-n* was the dichotomized expression of miRNAn.

$$\begin{array}{rcl}Prognostic\,Index & = & (EmiR-5010-3p\,\times \,\,-\,8.338526\,\times \,{10}^{-09})\\  &  & +\,(EmiR-5100\,\times \,\,-\,2.212730\,\times \,{10}^{-01})\\  &  & +\,(EmiR-656-3p\,\times \,\,-\,6.729683\,\times \,{10}^{-01})\\  &  & +\,(EmiR-671-3p\,\times \,\,-\,6.900286\,\times \,{10}^{-02})\end{array}$$

**Figure 1:**
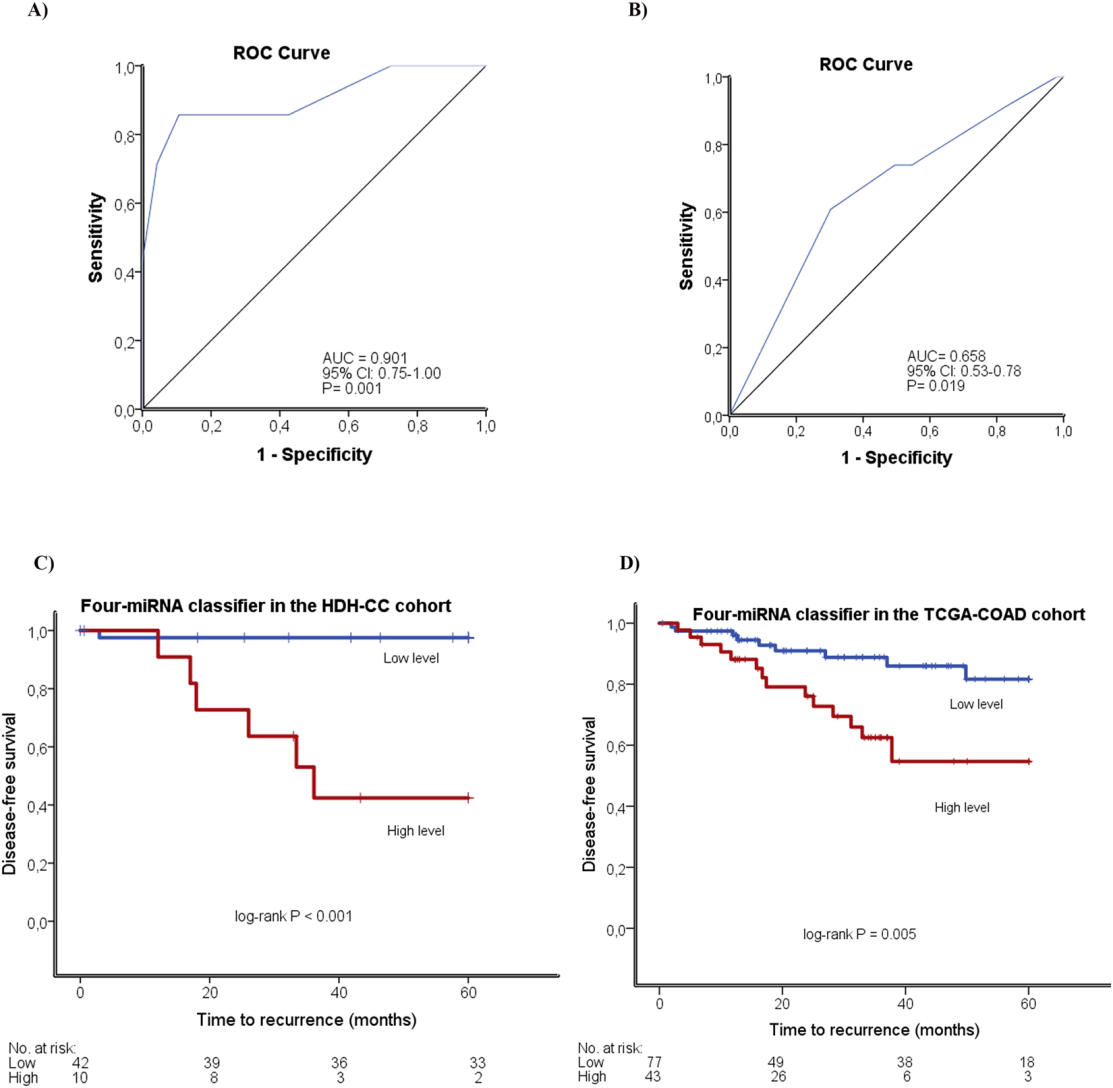
ROC curve analyses in the HDH-CC cohort (**A**) and in the TCGA-COAD cohort (**B**). Kaplan-Meier plots for the four-miRNA classifier in the HDH-CC cohort (**C**) and in the TCGA-COAD cohort (**D**).

The prognostic index was applied to all TNM-stage II colon cancer patients in the HDH-CC cohort and TCGA-COAD cohort. ROC-curve analyses were performed in order to generate the best cut-point of the prognostic index with optimal sensitivity and specificity at which low-risk patients can be distinguished from high-risk patients. The area under curve (AUC) in the ROC analyses for the HDH-CC and TCGA-COAD cohorts were statistically significant (AUC = 0.901, 95% CI: 0.75–1.00; P = 0.001; Fig. 1A and AUC = 0.658, 95% CI: 0.53–0.78; P = 0.0.19; Fig. 1B, respectively).

Eighty percent of the patients in the HDH-CC cohort and 64% of the patients in the TCGA-COAD cohort have a low risk of recurrence, according to our four-miRNA classifier (Table 1). We did not observe any statically significant associations between the level of the classifier and other clinicopathological features in the HDH-CC and TCGA-COAD cohort (Table 1).

### The prognostic value of the four-miRNA classifier in the HDH-CC cohort

Association between the four-miRNA classifier level and 5-year DFS were evaluated by performing univariate and multivariate Cox regression analyses. For the patients in the HDH-CC cohort, Kaplan-Meier analyses showed statistically significant better 5-year DFS for low versus high level of the four-miRNA classifier (97% vs 42%, P < 0.001; Fig. 1C). The sensitivity and specificity of the four-miRNA classifier for detecting recurrence were 86% and 90%, respectively. Multivariate Cox-regression analyses were not performed in the HDH-CC cohort because of the small number of events (recurrence, n = 7).

### Validation of the prognostic value of the four-miRNA classifier in an independent dataset (TCGA-COAD)

The prognostic value of the four-miRNA classifier was tested in the publically available TCGA-COAD dataset. In agreement with the findings for the HDH-CC cohort, high-risk patients had statistically significant shorter 5-year DFS than low-risk patients. Thus, patients with a low-level of the four-miRNA classifier have better 5-year DFS than patients with high-level, 82% versus 56% (P = 0.005; Fig. 1D). The sensitivity of the four-miRNA classifier was 61% and the specificity was 72% in this cohort. The multivariate Cox regression analyses showed that the four-miRNA classifier remained a statistically significant prognostic factor when adjusted for age, gender, MSI-status, total number of lymph node (TLN), and T-stage (HR 2.88; 95% CI: 1.20–6.94; P = 0.023; Table 2).

**Table 2:** Univariate and multivariate analyses for the four-miRNA classifier in the TCGA-COAD cohort.

	Univariate analyses			Multivariate analyses		
	**HR**	**95% CI**	**p-value**	**HR**	**95% CI**	**p-value**
Age (mean)			0.406			0.408
≤67.77	1			1		
>67.77	1.46	(0.59–3.56)		1.15	(0.57–3.97)	
Gender			0.141			0.239
Women	1			1		
Men	1.95	(0.80–4.74)		1.78	(0.68–4.54)	
MSI-status			0.425			0.589
MSS	1			1		
MSI	1.39	(0.61–3.17)		1.28	(0.51–3.23)	
TLN			0.740			0.745
≥12	1			1		
<12	0.78	(0.18–3.36)		0.78	(0.17–3.53)	
Tumor stage			0.114			0.073
T3	1			1		
T4	2.67	(0.79–9.07)		3.43	(0.89–13.2)	
Four-miRNA classifier			**0**.**007**			**0**.**023**
Low	1			1		
High	3.16	(1.36–7.33)		2.76	(1.15–6.62)	

HR = hazard ratio, CI = Confidence interval, TLN = total lymph node.

### Construction of nomogram based on the four-miRNA classifier

**Figure 2:**
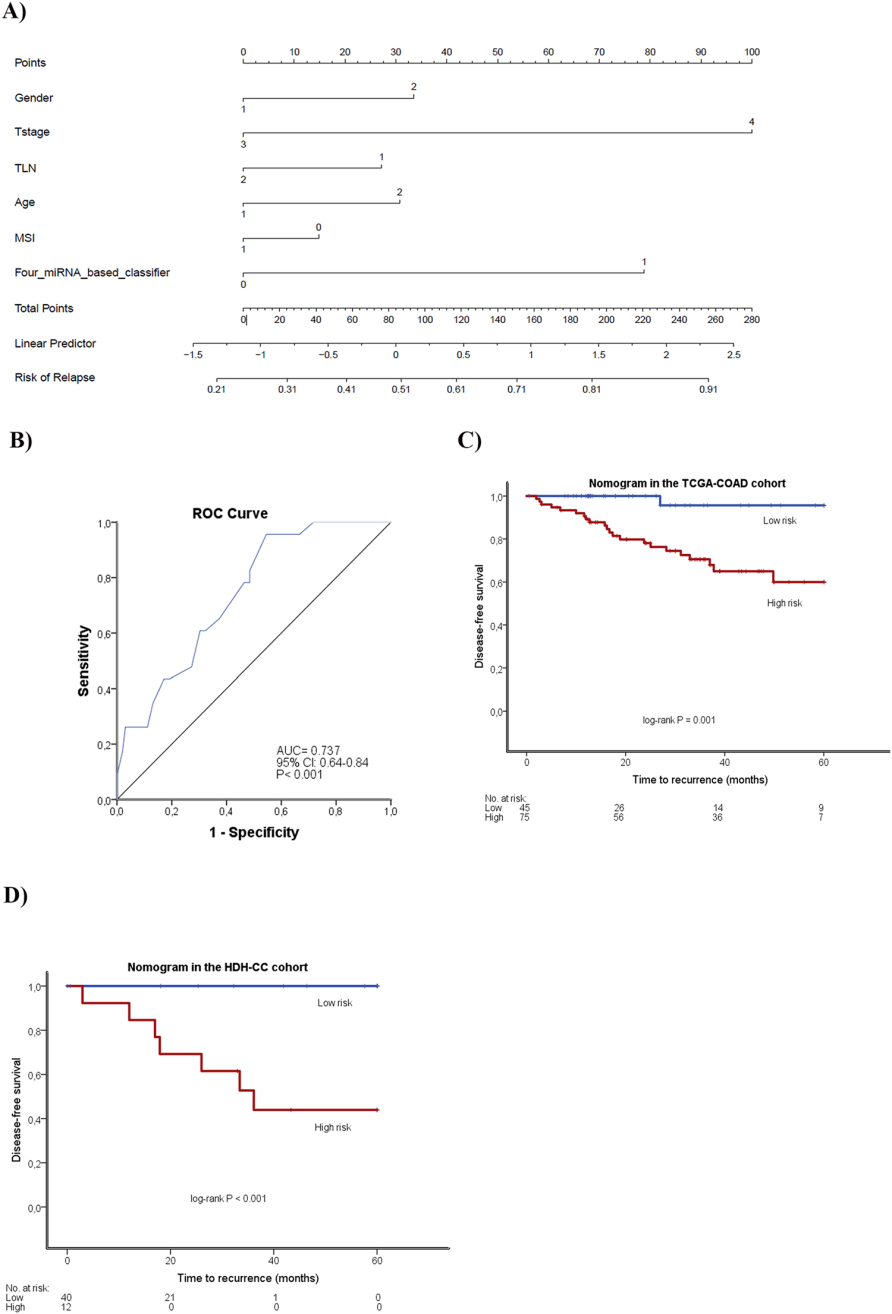
Nomogram based on gender (1 = women, 2 = men), Tstage (3 = T3, 4 = T4), TLN (<12 = 1, ≥12 = 2 Total Number of Lymph nodes), age (median age), MSI (0 = MSI, 1 = MSS) and the four-miRNA classifier (**A**). ROC curve analysis of Risk of Relapse calculated using the nomogram in the TCGA-COAD (**B**). Kaplan-Meier plots for 5-year DFS according to the nomogram in the TCGA-COAD (**C**) and HDH-CC (**D**) cohorts.

**Figure 3:**
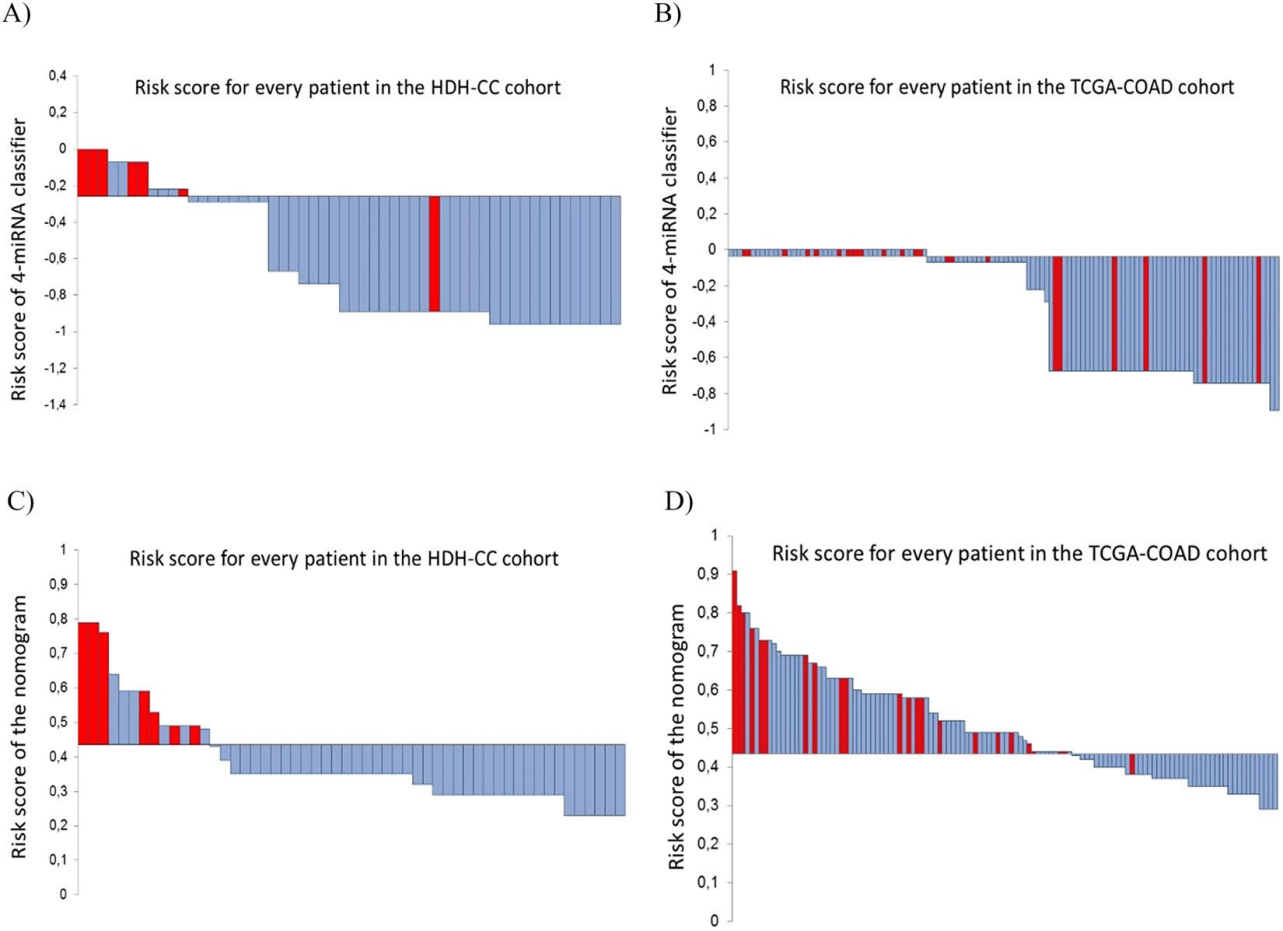
Risk score for every patient in the HDH-CC cohort (**A**) and in the TCGA-COAD cohort (**B**) based on the four-miRNA classifier alone. Risk score based on the nomogram in the HDH-CC cohort (**C**) and in the TCGA-COAD cohort (**D**). Red illustrates patients experiencing recurrence and blue illustrates patients with no recurrence.

We constructed a nomogram to predict the risk of recurrence in TNM-stage II colon cancer patients and to provide the clinician with a better estimation of the prognosis of the individual patient. The nomogram integrated age, gender, MSI-status, TLN, T-stage and the four-miRNA classifier (Fig. 2A). The nomogram identified T-stage and the four-miRNA classifier as the largest contributors to prognosis, followed by gender, age, TLN, and MSI. We calculated the risk score by adding up the scores from each variable, calculated the Total Point score and drew a straight line down to determine the estimated risk of recurrence. The prognostic accuracy of the nomogram was assessed using ROC analyses (Fig. 2B). The cut-point for the risk of recurrence determined by ROC curve analyses resulted in a sensitivity and specificity of 96% and 46%, respectively in the TCGA-COAD cohort. The KM plot of the TCGA-COAD cohort showed that the stratification of the patients in low- and high risk groups is statistically significant (P = 0.001; Fig. 2C). Further, the nomogram was tested in the HDH-CC cohort, using the same cut-point as for the TCGA-COAD cohort. The results showed that the nomogram identified the patients experiencing recurrence with a sensitivity of 100% and a specificity of 85% (P < 0.001; Fig. 2D). Waterfall plots were generated in order to illustrate the risk score for every patient in both cohorts based on the four-miRNA classifier (Fig. 3A,B), and based on the nomogram (Fig. 3C,D).

## Discussion

Clinical trials have shown that although 20% of TNM-stage II colon cancer patients are at high risk of recurrence, the benefit of current adjuvant chemotherapy treatment is small in TNM-stage II colon cancer patients as a whole. In the current study, we constructed and validated a four-miRNA classifier as a prognostic biomarker to predict recurrence in TNM-stage II colon cancer patients. The four-miRNA classifier was developed using LASSO cox regression in the HDH-CC cohort (n = 54) and validated in TCGA-COAD cohort (n = 122). The classifier successfully discriminated between patients at high- and low risk of recurrence. High-risk patients had statistically significant shorter 5-year DFS than low-risk patients. Accordingly, a low level of the four-miRNA classifier was associated with better survival in both HDH-CC and TCGA-COAD cohort. The sensitivity and specificity of the four-miRNA classifier appears to be better in the HDH-CC cohort (86% and 90%, respectively) than in the TCGA-COAD cohort (61% and 72%, respectively). The 5-year DFS in the TCGA-COAD is lower than in the HDH-CC cohort (72% versus 85%, respectively), which may have an impact on the differences. The classifier remained statistically significant in multivariate analyses in the TCGA-COAD cohort. Because of the relative small number of patients and few recurrences in the HDH-CC cohort, we did not perform multivariate analyses in this cohort. Nomograms have been developed to evaluate several significant clinicopathological risk factors. We constructed a nomogram including the four-miRNA classifier to develop a practical method for clinicians to predict the probability of TNM-stage II colon cancer recurrence. Compared to the classifier alone, the prognostic nomogram improved the sensitivity from 86% to 100% in the HDH-CC cohort, and from 61% to 96% in the TCGA-COAD cohort. On the other hand, the nomogram does not improve the specificity; from 90% to 85% in the HDH-CC and from 72% to 46% in the TCGA-COAD cohort.

Therefore, we argue that the four-miRNA classifier alone is able to predict the probability of recurrence; however, we can not exclude the possibility that the nomogram could be more robust than the classifier alone for identifying patients at risk for recurrence in other colon cancer patient cohorts.

In clinical practice, histopathological and clinical factors are used to determine prognosis and treatment in colon cancer^23^. However, histologic tumor staging has not been consistently associated with increased risk of recurrence in TNM-stage II colon cancer^31^. Further, MMR or MSI status are documented as the most validated and important prognostic biomarkers in TNM-stage II colon cancer patients^12,13^. Patients with MSI have low recurrence rates and good outcomes without adjuvant chemotherapy^17,21^. A previous study describes different miRNA expression pattern in deficient and proficient MMR^32^. In the present study, there was no statistically significant association between MMR status and the four-miRNA classifier (Table 1). In the HDH-CC cohort, 13 out of 15 patients (87%) with deficient MMR were classified as low risk, and 27 out of 35 patients (77%) with proficient MMR were also classified as low risk patients (Supplementary Table 1). Therefore, the good prognostic value of the four-miRNA classifier is not associated with MMR-status in this study. Further, the four-miRNA classifier was not able to classify patients into low- and high risk groups in TNM-stage III colon cancer patients in the two cohorts (Supplementary Fig. 3).

Therefore, the prognostic value of the four-miRNA classifier is specific for TNM-stage II colon cancer patients.

In addition, several miRNA studies have identified miRNA signatures as prognostic biomarkers in colon cancer cancer^28–30,33^. The results of the tissue-based miRNA signature studies are based on microarray technology or RT-qPCR. Earlier, we have identified a 16-miRNA signature as a prognostic biomarker for TNM-stage II and III colon cancer patients. The study was based on RT-qPCR and the panel we used included only 83 selected, cancer related miRNAs^30^. The current study is based on next-generation sequencing which has the advantage of global miRNA analysis, higher sensitivity and the ability to detect novel miRNAs^34^. Interestingly, the four miRNAs included in the classifier have not previously been documented as prognostic markers in colon cancer. However, there are several studies linking these four miRNAs to other cancer types or tumor models. One previous study found an increased level of miR-5100 in non-small-cell lung cancer and its association with poor prognosis was documented^35^. A functional study showed that this miRNA promotes tumor growth as a result of targeting Rab6, a protein involved in membrane traffic from the Golgi apparatus to the endoplasmatic reticulum and exocytosis^36^. Downregulation of miR-656 in cluster with miR-379 were found in multiple cancers in a genome-wide study^37^. The miR-379/miR-656 cluster was also identified as a tumor suppressor locus in adult medulloblastomas^38^. Another study identified an eleven-miRNA signature including miR-671 as a prognostic biomarker for a subtype of kidney cancer with wild type BRCA1 associated protein-1 (BAP-1)^39^. Regarding miR-5010, no functional or clinical studies have been published yet; however, a number of genes are predicted to be targets of this miRNA with high relevance to tumor progression, among others, member of RAS oncogene family (RAB9A), APC membrane recruitment protein 1 (AMER1), beta-catenin interacting protein 1 (CTNNBIP1), Ras-related GTP binding D (RRAGD), etc. (miRDB; www.mirdb.org and TargetMiner; www.isical.ac.in).

In summary, we constructed a classifier based on expression of four miRNAs in fresh frozen tumor tissue from TNM-stage II colon cancer patients. The classifier is able to distinguish between high- and low-risk patients, which enables the clinicians to identify the patients that need to be followed closely in order to detect any recurrence at an early stage and administrate chemotherapy to patients at high-risk of recurrence.

## Patients and Methods

### Patient cohorts

This study was approved by The Regional Ethics Committee West and the Data Inspectorate for National Registries. The patients signed the informed consent and the original clinical trial was registered at U.S National Institute of Health (ClinicalTrials.gov, NCT00963352). All methods were conducted in accordance with approved guidelines and regulations.

Three hundred and ninety six TNM stage I-IV colon cancer patients had undergone surgical resection for primary tumor between January 2007 and December 2011 at Haraldsplass Deaconess Hospital (HDH-CC; Supplementary Fig. 1)^40^. We performed NGS of miRNAs in 128 TNM-stage I-IV tumor samples and 10 adjacent normal colon samples with available fresh frozen tissue. Out of 128 patients, 54 patients were classified as TNM-stage II. None of these patients received radiotherapy or adjuvant chemotherapy^41^. The patients had a regular check-up, which included clinical examination, chest x-ray, or CT, abdominal ultrasound or CT and CEA blood testing until the fifth year of the follow-up after surgery. Colonoscopy was performed three years after the date of surgery. The four mismatch repair proteins were assessed by immunohistochemistry using MLH1 (1:60, DAKO, M3640, clone ES05), MSH-2 (1:300, Biocare Medical, CM219B, clone FE11), MSH6 (1:50, DAKO, M3646, clone EP49) and PMS2 (1:50, DAKO, M3647, clone EP51). Epitope retrieval (HIER) was performed in TE-buffer pH9, and the detection kit used was MACH3 HRP-Polymer (Biocare Medical).

For validation of our findings, we downloaded miRNA-sequencing data from the TCGA-COAD cohort from NIH National Cancer Institute Genomic Data Commons Data Portal (The Cancer Genome Atlas; https://cancergenome.nih.gov/). We included all 122 TNM-stage II colon cancer patients for which miRNA sequencing results and the requested clinicopathological and survival information were available (Supplementary Fig. 2). MSI status for the TCGA-COAD patients was downloaded from the TCGA Data Portal (NIH). According to information available from the TCGA consortium, a panel of four mononucleotide repeat loci (BAT25, BAT26, BAT40, and transforming growth factor receptor type II) and three dinucleotide repeat loci (CA repeats in D2S123, D5S346, and D17S250) were assessed^42^. The patients were classified as MSI-high if more than 40% of the markers were altered, and patients with lower than 40%-altered marker were classified as MSS or MSI-low.

### RNA extraction

Total RNA from the fresh frozen tissue samples was extracted using the miRNeasy, Mini kit (QIAGEN) according to the manufacturer’s protocol. Approximately 30 mg of each sample was homogenized in 700 µl QIAzol Lysis Reagent using Tissuelyzer (QIAGEN) for 10 minutes at 25 Hz. The homogenate was processed according to the manufacturer’s instruction and the total RNA was purified using a protocol where DNase treatment was included. RNA concentrations were measured by NanoDrop and the quality of RNA was measured by Agilent RNA Bioanalyzer (Agilent RNA 6000 Nano Assay protocol-edition April 2007). The RNA samples were stored at −80 °C until use.

### MicroRNA sequencing

The Illumina TruSeq RNA sample Prep Kit (Illumina, Inc., San Diego, CA, USA) was used to construct cDNA libraries for sequencing. In brief, one µg of the total RNA was used to sequentially ligate 3′ and 5′ adapters to the ends and reverse transcribe to generate cDNA. The cDNA was amplified using 15 PCR cycles. The amplified cDNA was purified and quantified on a 6% Novex TBE gel. The quality control was performed using Agilent Technologies 2100 Bioanalyzer. The resulting pooled libraries were sequenced on Illumina HighSeq4000 (Illumina, Inc., San Diego, CA, USA).

### Processing of the sequencing data

The raw miRNA sequences were output in FASTQ format. The raw reads were initially filtered for reads containing adapter sequences and low-quality sequences. miRDeep2 were used to map the sequences to miRNAs and quantify expression of the specific miRNAs^43^. The counts were then processed by edgeR Bioconductor package using the Trimmed Mean of M (TMM) method for normalization^44^.

### Statistical analysis

The R software (version 3.3.2) and the “glmnet” package were used to perform the LASSO regression model analysis in the HDH-CC cohort. The penalized Cox regression model was determined through 200-times cross validation^45,46^. Based on the optimal lambda value, a panel of four miRNAs with associated coefficients was identified as the best prognostic marker for recurrence among the TNM-stage II colon cancer patients in the HDH-CC cohort. The risk score for each patient was calculated based on the dichotomized expression of each prognostic miRNA using X-tile plots (software version 3.6.1, Yale University School of Medicine, New Haven, CT, USA) and its associated regression coefficient^47^. The patients in the HDH-CC and TCGA-COAD cohort were split into low-risk or high-risk groups according to the receiver operating characteristic (ROC) curve. The disease- free survival (DFS) time of patients were from date of surgery to the time of recurrence or colon cancer related death before documented recurrence. Deaths of other causes were censored and maximum follow-up time was 5-years. Survival differences among the risk groups were analyzed by the Kaplan-Meier method with log-rank test, and the multivariate survival analyses were performed using Cox-regression model. ROC-curves and survival analyses were performed using SPSS (IBM SPSS Statistics, version 24) and all P-values considered statistically significant if P < 0.05. We designed a nomogram to provide the overall probability of risk of recurrence using the “rms” package of R software (version 3.3.2)^48^. The nomogram was generated in the TCGA-COAD cohort because the number of patients and recurrent events in this cohort were larger than in the study cohort. ROC curves were used to assess the accuracy of the nomogram in the TCGA-COAD - and the HDH-CC cohort.

## Electronic supplementary material

Supplementary information
