# Supplementary material for: A four-microRNA classifier as a novel prognostic marker for tumor recurrence in stage II colon cancer
Source: Sci Rep. 2018 Apr 18;8:6157. doi: 10.1038/s41598-018-24519-4 (PMC5906690; doi:10.1038/s41598-018-24519-4)
Supplement: Supplementary file 1 — Supplementary information [file 41598_2018_24519_MOESM1_ESM.pdf]

Supplementary information

Manuscript title: A four-microRNA classifier as a novel prognostic marker for tumor recurrence in stage II colon cancer

Author list:

Author ID page

1. Havjin Jacob
   MSc.
   Department of Clinical Science, Faculty of Medicine, University of Bergen, Bergen, Norway

2. Luka Stanisavljevic
   MD. PhD
   Department of Oncology and Medical Physics, Haukeland University Hospital, Bergen, Norway

3. Kristian Eeg Storli
   MD. PhD
   Department of Surgery, Haraldsplass Deaconess Hospital, Bergen, Norway

4. Kjersti Elvestad Hestetun
   MD.
   Department of Clinical Science, Faculty of Medicine, University of Bergen, Bergen, Norway

5. Olav Dahl
   MD. PhD
   Department of Oncology and Medical Physics, Haukeland University Hospital, Bergen, Norway

6. Mette Pernille Myklebust
   MSc. PhD.
   Department of Oncology and Medical Physics, Haukeland University Hospital, Bergen, Norway

Supplementary Figure 1: Flowchart of the HDH-CC cohort.

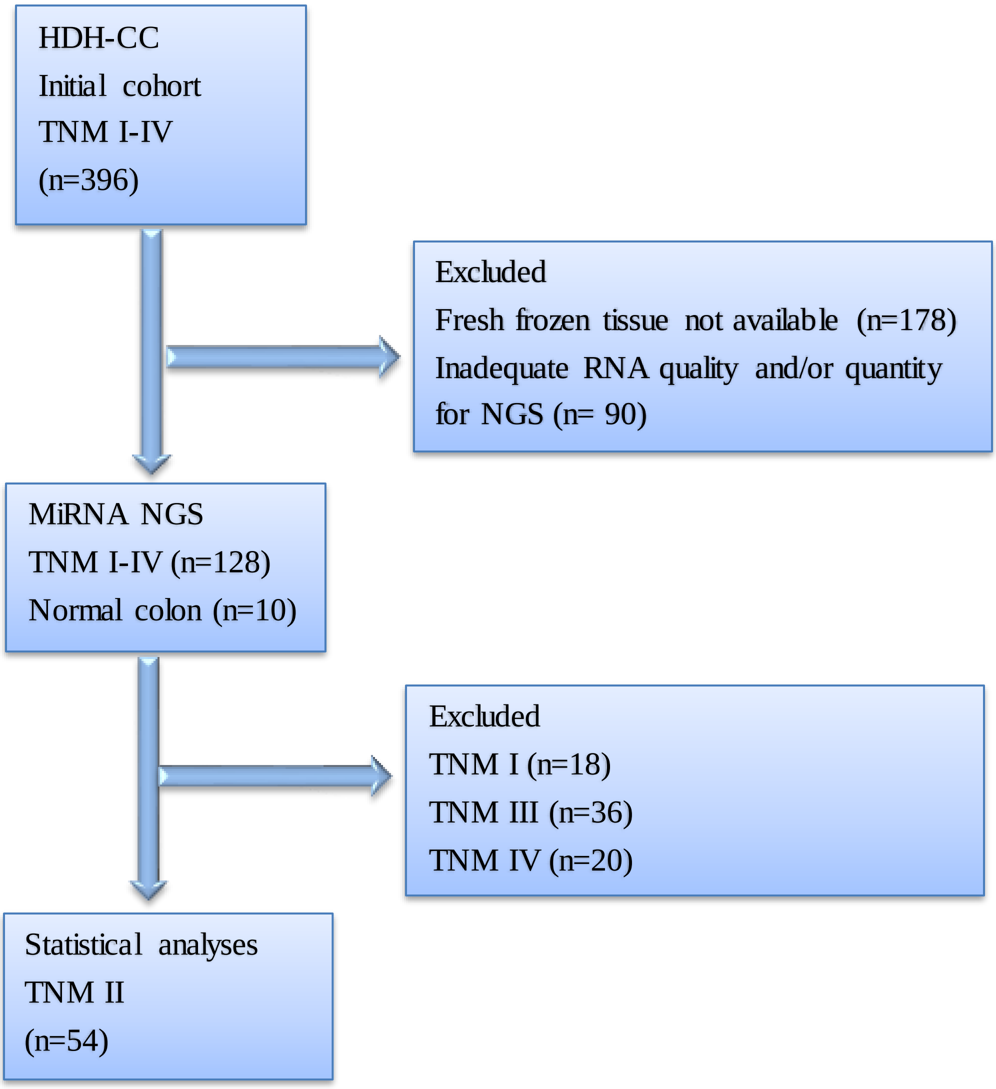

Supplementary Figure 2: Flowchart of the TCGA-COAD cohort.

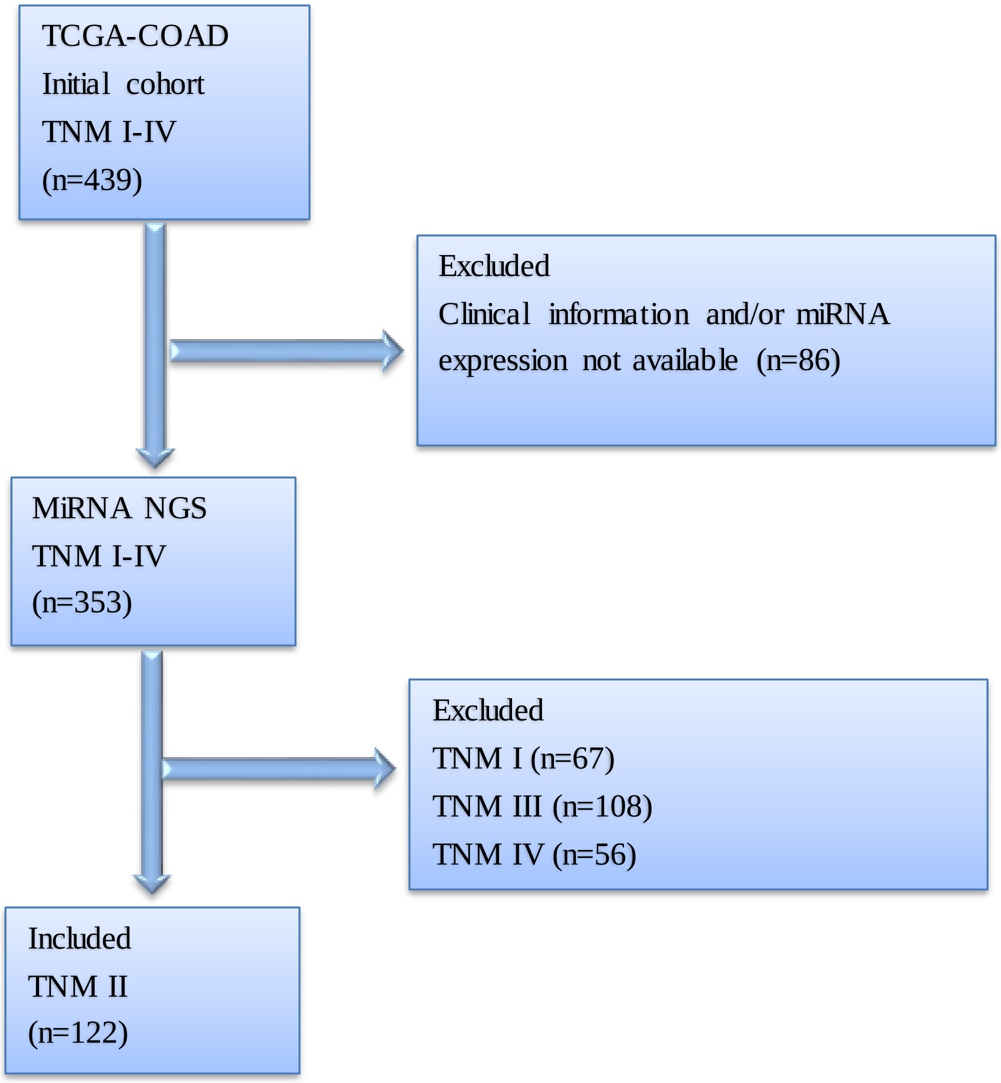

Supplementary Figure 3: Kaplan-Meier plots for the four-miRNA classifier in TNM-stage III colon cancer patients in the HDH-CC cohort (A) and TCGA-COAD cohort (B).

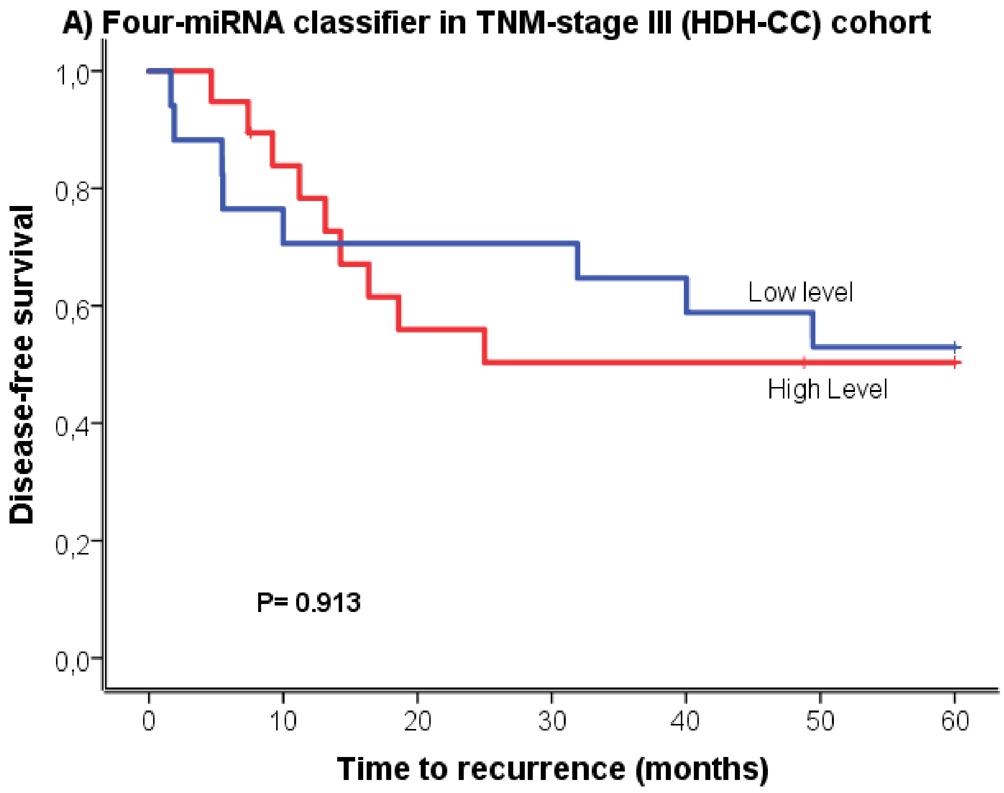

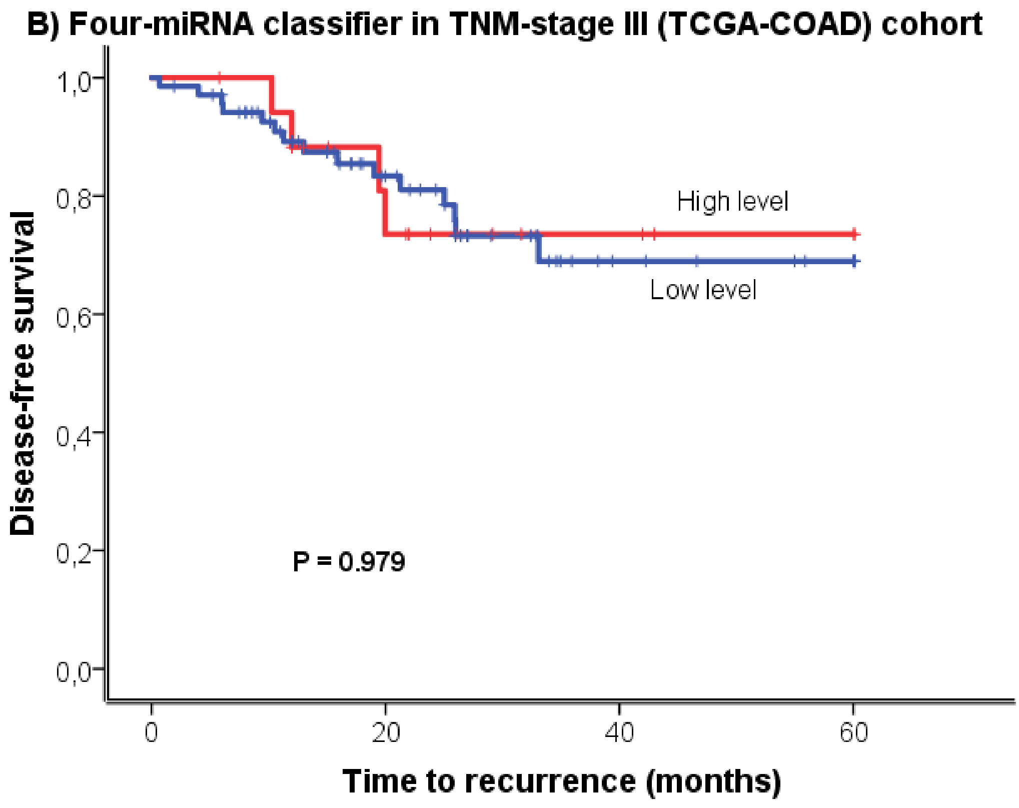

Supplementary Table 1: The association between MMR-status and four-miRNA classifier in the HDH-CC cohort.

Patient_ID	Recurrence	MMR-status	four-miRNA classifier
176	No	MMR-def	High
265	No	MMR-def	High
82	No	MMR-def	Low
116	No	MMR-def	Low
117	No	MMR-def	Low
123	No	MMR-def	Low
164	No	MMR-def	Low
173	No	MMR-def	Low
190	No	MMR-def	Low
204	No	MMR-def	Low
264	No	MMR-def	Low
271	No	MMR-def	Low
272	No	MMR-def	Low
292	No	MMR-def	Low
361	No	MMR-def	Low
91	No	MMR-prof	High
118	No	MMR-prof	High
122	No	MMR-prof	High
174	Yes	MMR-prof	High
208	Yes	MMR-prof	High
211	Yes	MMR-prof	High
217	Yes	MMR-prof	High
320	Yes	MMR-prof	High
340	Yes	MMR-prof	High
77	No	MMR-prof	Low
94	No	MMR-prof	Low
99	No	MMR-prof	Low
105	No	MMR-prof	Low
115	No	MMR-prof	Low
119	No	MMR-prof	Low
124	No	MMR-prof	Low
136	No	MMR-prof	Low
154	No	MMR-prof	Low
171	No	MMR-prof	Low
183	No	MMR-prof	Low
188	No	MMR-prof	Low
196	No	MMR-prof	Low
209	No	MMR-prof	Low
223	No	MMR-prof	Low
224	No	MMR-prof	Low
231	No	MMR-prof	Low

267	No	MMR-prof	Low
268	No	MMR-prof	Low
293	No	MMR-prof	Low
305	No	MMR-prof	Low
308	No	MMR-prof	Low
309	No	MMR-prof	Low
330	No	MMR-prof	Low
332	No	MMR-prof	Low
362	No	MMR-prof	Low
197	No	Not determined	High
346	Yes	Not determined	High
97	No	Not determined	Low
219	No	Not determined	Low
